# Unified Chassis Control of Electric Vehicles Considering Wheel Vertical Vibrations

**DOI:** 10.3390/s21113931

**Published:** 2021-06-07

**Authors:** Xinbo Chen, Mingyang Wang, Wei Wang

**Affiliations:** Institute of Intelligent Vehicles, School of Automotive Studies, Tongji University, No. 4800 Cao’an Highway, Shanghai 201804, China; austin_1@163.com (X.C.); wangmingyang@sina.cn (M.W.)

**Keywords:** electric vehicle, unified chassis control, unsprung mass

## Abstract

In the process of vehicle chassis electrification, different active actuators and systems have been developed and commercialized for improved vehicle dynamic performances. For a vehicle system with actuation redundancy, the integration of individual chassis control systems can provide additional benefits compared to a single ABS/ESC system. This paper describes a Unified Chassis Control (UCC) strategy for enhancing vehicle stability and ride comfort by the coordination of four In-Wheel Drive (IWD), 4-Wheel Independent Steering (4WIS), and Active Suspension Systems (ASS). Desired chassis motion is determined by generalized forces/moment calculated through a high-level sliding mode controller. Based on tire force constraints subject to allocated normal forces, the generalized forces/moment are distributed to the slip and slip angle of each tire by a fixed-point control allocation algorithm. Regarding the uneven road, H∞ robust controllers are proposed based on a modified quarter-car model. Evaluation of the overall system was accomplished by simulation testing with a full-vehicle CarSim model under different scenarios. The conclusion shows that the vertical vibration of the four wheels plays a detrimental role in vehicle stability, and the proposed method can effectively realize the tire force distribution to control the vehicle body attitude and driving stability even in high-demanding scenarios.

## 1. Introduction

With the growing concern about pollution, energy shortage, and also fast development of electric propulsion technologies, modern vehicles are increasingly electrified. From the driver’s point of view, an adequate response in critical driving conditions is still a challenging task for non-professional drivers. Therefore, electric or electromechanical systems, featuring energy regeneration capability and fast response, are readily developed and applicated to improve the vehicle dynamics, from the aspects of comfort, stability, safety, maneuverability, and driver’s feeling, especially in adverse driving situations.

In order to enhance the driving stability and the overall dynamic performance, a vehicle may be equipped with multi-actuators, which can be classified into three categories as active torque distribution [1], active steering [2], and active suspension control [3]. Among all possible actuators, ABS-based differential braking has received the most attention since it can be executed on almost all vehicles regardless of powertrain configuration, known as the traditional ESC system. In 2003, the active front steering technology was developed and recognized as a supplemental approach to generate desired yaw moment without braking, ensuring enhanced vehicle stability even in high-speed conditions. However, the differential braking systems reduce the driving speed, which may conflict with the driver’s intention during acceleration scenarios. Concerning this defect, active torque distribution was implemented by two actuation methods: torque vectoring [4] and individual motors. The corresponding vehicle stability control method was called direct yaw control.

In most vehicle control approaches, different control logic based on various actuators are always separately synthesized and locally tuned without considering the interaction among them, which may lead to sub-optimal or conflicting control efforts. Nowadays, extensive research has been carried out on the coordination of active steering and independent torque control. Furthermore, the integration with active suspension systems provides a new research field under the name of global chassis control [5,6,7] or UCC (Unified Chassis Control) [8,9,10]. In general, most integrated control algorithms were developed using two different approaches concerning the actuators hierarchy: (1) In the first method, a supervisor with a higher command hierarchy was used to monitor vehicle states and coordinate different sub-controllers. As illustrated, sometimes the active steering was only considered if the differential braking system exceeded its limits or before the ESC was activated [11]. In [12], the coordination of active front steering and ESC was investigated using a rule-based method according to the different value ranges of lateral acceleration. (2) The second UCC category treated the overall control structure as two levels. In the upper-level, desired yaw moment was computed, then in the lower-level, the moment was distributed into tire forces [8,13].

From a control point of view, a variety of problems arise from the UCC system synthesis, such as multiple input–multiple output control design, system robustness, and non-linearity. Many researchers have tried to solve these challenges from the standpoints of reference track following and control optimization. Fuzzy logic was applied for an intermediate layer of a UCC method [14]. The sliding mode control technique, which possesses good robustness, was used to cope with system uncertainties [7,15]. In [11,16], control objectives were achieved in a linear parameter varying robust framework by providing a solution to the linear matrix inequality problem. The H∞-based observer is also designed for fault estimation and fault-tolerant control [17,18]. Model prediction control becomes a hotspot due to significant development on online computational devices [19,20,21]. Another challenge stemmed from how to achieve the constrained optimal allocation problems in systems with redundancy. In [9], the desired yaw moment distribution was algebraically solved by Karush–Kuhn–Tucker Conditions. Combined with the desired target following, energy minimization, and tire force saturation, the Holistic Cornering Control architecture was introduced [22,23]. Under this framework, the gain optimization was designed based on linear matrix inequality and genetic algorithm techniques. In [24], the redundant actuator allocation problem was investigated with pseudo-inverse and accelerated fix-point iterative algorithms. Since the tire normal load restraints the boundary of longitudinal and lateral force, the vertical force control indirectly influences the vehicle dynamics in the motion plane. The research in [25] has shown the possible benefits to be attained by modulating normal force through active suspension control during cornering maneuvers. Regarding the multiple targets of vehicle yaw performance and attitude, the integration control method involving suspensions and braking actuators was investigated in [26,27]. However, the integrated suspension control does not consider the tire contact stability.

Different from traditional vehicle chassis structures, the layout with four in-wheel motors is especially suitable for high-performance and off-road vehicles. However, this layout inevitably introduces a larger unsprung mass. With the development of in-wheel motor technology, it becomes a common judgment that the introduced unsprung mass aggravates the wheel vibration and vehicle ride comfort [28]. Especially when the vehicle is running over an uneven road, one or more of the wheels might jump, and the tires will lose adhesion stochastically, which leads to the unbalance of driving/braking torques among four wheels and even makes the vehicle encounter instability situations. This effect occurs not only for in-wheel drive electric vehicles but also for common vehicles. Till now, little research can be found in this area. Vos et al. [29] verified that the vertical accelerations caused by bad roads could increase the roll and pitch movement ranges. Zhang et al. [30] proposed the inference that the unsprung mass plays a different role in the rollover motion between a flat road and an uneven road, and verified it with typical situations. Tan et al. [31] investigated the negative effect of wheel motor vibration on vehicle anti-rollover performance. In addition, the tire contact stability has been given little attention in existing UCC designs.

The purpose of this work is to present a unified control framework with a particular focus on wheel vertical vibrations for enhanced tire contact stability. More specifically, we treat the multi-objective active suspension design problem as a robust tracking problem to achieve desired body attitude and normal forces simultaneously. Besides, a reconfigurable control allocation (CA) method is used to deal with the problematic condition of tire contact force loss due to an uneven road or tire blow-out emergency. Evaluation of the overall system was accomplished by simulation testing with a full-vehicle CarSim model under different test scenarios.

The remaining sections of this paper are organized as follows: Section 2 briefly presents the overall system modeling. In Section 3, nonlinear controller design and reconfigurable CA approach are presented, and the synthesis of the normal force controller considering different targets is described, followed by Section 4, which examines the closed-loop vehicle dynamics performance under different test scenarios through simulation results. Section 5 provides some concluding remarks.

## 2. System Modeling

### 2.1. Nonlinear Vehicle Model

**Figure 1:**
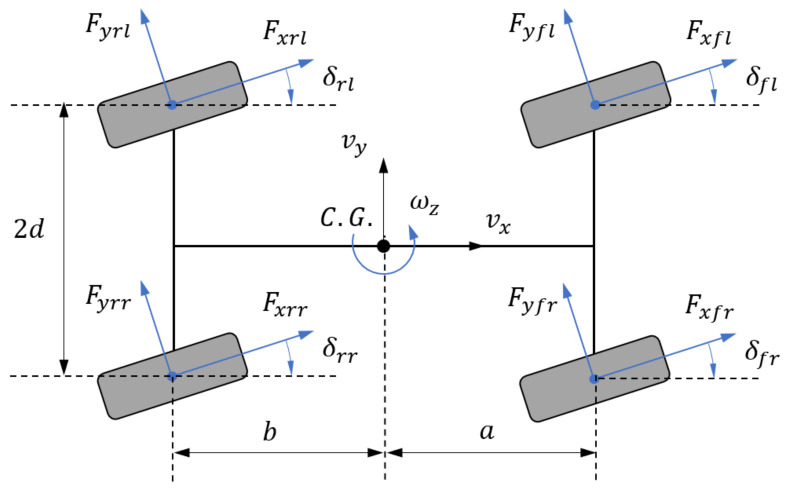
Vehicle planar motion model.

In this paper, both the chassis planar motion (longitudinal, lateral, and yaw) and the spatial motion (vertical, roll, and pitch) are considered. Thus, the vehicle body motion is treated as a rigid body with six degrees of freedom. Figure 1 shows the vehicle planar motion model. The dynamics equations of planar motions can be written as

(1) $$\left\{\begin{array}{l} m\left({\dot{v}}_{x}-v_{y}\omega_{z}\right)+m_{s}e_{r}{\dot{\omega }}_{z}\varphi =F_{X}-F_{r}\\ m\left({\dot{v}}_{y}+v_{x}\omega_{z}\right)-m_{s}e_{r}\ddot{\varphi }=F_{Y}\\ I_{zz}{\dot{\omega }}_{z}=M_{z} \end{array}\right.,$$

with *m* as the vehicle mass (including the sprung mass *m_s_* and unsprung mass *m_u_*) and *I_zz_* as the moment of inertia along Z-axis. The vehicle states are defined as the body motion velocity in three directions, namely (*v_x_ v_y_ ω_z_*), with the vehicle coordinate system fixed at the vehicle Center of Gravity (CG). *e_r_* denotes the distance of CG from the rolling center and *φ* is the rolling angle. The resultant forces exerted on the vehicle are defined as *F_X_*, *F_Y_*, and *M_Z_*, with the following expressions

(2) $$\left\{\begin{array}{l} F_{X}=\sum F_{Xi},\;i\in Q:=fl,fr,rl,rr\\ F_{Y}=\sum F_{Yi},\;i\in Q:=fl,fr,rl,rr \end{array}\right.,$$

(3) $$M_{Z}=a\left(F_{Yfl}+F_{Yfr}\right)-b\left(F_{Yrl}+F_{Yrr}\right)+d\left(F_{Xfr}+F_{Xrr}-F_{Xfl}-F_{Xrl}\right)$$

*F_r_* stands for the total longitudinal resistance. *F_Xi_* and *F_Yi_* are the tire forces under the vehicle coordinate system, whereas the longitudinal and lateral forces of a single tire under the tire coordinate system are expressed as *F_xi_* and *F_yi_*. Their relationship is described as follows.

(4) $$\left[\begin{array}{c} F_{Xi}\\ F_{Yi} \end{array}\right]=\left[\begin{array}{cc} \cos\delta_{i} & -\sin\delta_{i}\\ \sin\delta_{i} & \cos\delta_{i} \end{array}\right]\left[\begin{array}{c} F_{xi}\\ F_{yi} \end{array}\right],i\in Q:=fl,fr,rl,rr$$

**Figure 2:**
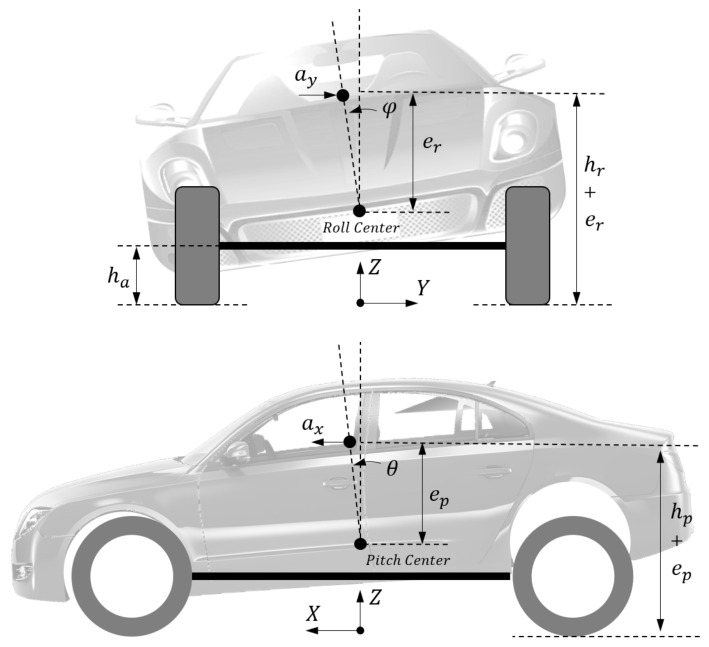
Vehicle roll and pitch motion.

Considering the assumption that the vehicle has a four-wheel independent steering system, *δ_i_* stands for the steering angle of a given wheel with the subscript representing the position which is controlled by every steering actuator. In Figure 2, the vehicle spatial motion models are illustrated. Concerning the unsprung mass dynamics, we treat the vehicle as a whole system under the influence of lateral and longitudinal accelerations.

Based on the reference direction in Figure 2, if we treat the sprung/unsprung mass as a whole system, the roll and pitch dynamic equations can be respectively expressed as

(5) $$I_{xx}\ddot{\varphi }=M_{\varphi }+a_{y}m_{s}\left(h_{r}+e_{r}\right)+m_{s}g\varphi e_{r}+a_{y}m_{u}h_{a},$$

(6) $$I_{yy}\ddot{\theta }+a_{x}m_{s}\left(h_{p}+e_{p}\right)+a_{x}m_{u}h_{a}=M_{\theta }+m_{s}g\theta e_{p},$$

where, *I_xx_* and *I_yy_* are the principal moments of inertia of the vehicle body part along X and Y axis, respectively. (*h_r_* + *e_r_*) and (*h_p_* + *e_p_*) represent the heights of the rolling center and pitching center, respectively, with *e_r_* as the distance between vehicle CG and rolling center and *h_p_* as the distance between vehicle CG and pitching center. *h_a_* represents the height of the unsprung mass center. The vehicle longitudinal/lateral accelerations can be calculated as,

$$a_{x}={\dot{v}}_{x}-v_{y}\omega_{z},\;a_{y}={\dot{v}}_{y}+v_{x}\omega_{z}$$

These values are assumed to be available through a sensing system. Here, we ignore the mass of suspension links and assume that the unsprung mass CG is located in the wheel center. *M_θ_* and *M_φ_* denote the moments generated by the load transfer among vertical tire forces, and we describe this relationship in the form of

(7) $$\left\{\begin{array}{l} F_{Zfl}={\bar{F}}_{Zfl}-\frac{M_{\theta }}{2(a+b)}+\frac{M_{\varphi }}{4d},F_{Zfr}={\bar{F}}_{Zfr}-\frac{M_{\theta }}{2(a+b)}-\frac{M_{\varphi }}{4d},\\ F_{Zrl}={\bar{F}}_{Zrl}+\frac{M_{\theta }}{2(a+b)}+\frac{M_{\varphi }}{4d},F_{Zrr}={\bar{F}}_{Zrr}+\frac{M_{\theta }}{2(a+b)}-\frac{M_{\varphi }}{4d}, \end{array}\right.$$

where, *F_Zi_* are static normal tire forces and *F_Zi_* are desired tire normal forces, which can be achieved using four independent active suspension actuators.

### 2.2. Nonlinear Tire Model

We applied Pacejka’s Magic Formula tire model [32] to describe the nonlinear characteristics of tires, which is an empirical approach and can be effectively matched with experimental data. The longitudinal force, lateral force, and self-aligning moment of tire can be expressed by the following unified form with different parameter sets.

(8) $$\left\{\begin{array}{l} y\left(x\right)=D\sin\left\{C\arctan\left[Bx-E\left(Bx-\arctan Bx\right)\right]\right\}\\ Y\left(X\right)=y(x)+S_{v}\\ x=X+S_{h} \end{array}\right.,$$

where *Y* represents the tire force that could be the longitudinal/lateral tire force or self-aligning moment. *X* denotes the model input, which corresponds to the tire slip or the slip angle. *S_v_* and *S_h_* correspond to the vertical and horizontal bias, respectively. Coefficients *B*, *C*, *D*, and *E* are the stiffness, shape, peak, and curvature factors, respectively. When considering the coupling of lateral and longitudinal force, we usually neglect the curvature factor for simplicity, and then the following relations hold approximately.

(9) $$F_{xi}=\frac{\sigma_{xi}}{\sigma_{i}}F_{x0i},F_{yi}=\frac{\sigma_{yi}}{\sigma_{i}}F_{y0i}$$

*F_x_*_0_*_i_* and *F_y_*_0_*_i_* can be calculated by applying their corresponding magic formulas, with *σ_xi_* as the longitudinal tire slip expressed below and *σ_yi_* as the lateral tire slip.

(10) $$\left\{\begin{array}{l} \sigma_{xi}=\frac{R_{i}\omega_{i}-v_{wxi}}{v_{wxi}},\;\mathrm{during}\;\mathrm{braking}\\ \sigma_{xi}=\frac{R_{i}\omega_{i}-v_{wxi}}{R_{i}\omega_{i}},\;\mathrm{during}\;\mathrm{acceleration} \end{array}\right.,$$

where, *v_wxi_* expresses the velocity at tire center in the tire forward direction. According to the mentioned vehicle planar model, the slip angles of each wheel are

(11) $$\left\{\begin{array}{l} \alpha_{fl}=-\delta_{fl}+\arctan\left(\frac{v_{y}+\dot{\psi }a}{v_{x}-\dot{\psi }d}\right)\approx -\delta_{fl}+\frac{v_{y}+\dot{\psi }a}{v_{x}-\dot{\psi }d}\\ \alpha_{fr}=-\delta_{fr}+\arctan\left(\frac{v_{y}+\dot{\psi }a}{v_{x}+\dot{\psi }d}\right)\approx -\delta_{fl}+\frac{v_{y}+\dot{\psi }a}{v_{x}+\dot{\psi }d}\\ \alpha_{fr}=-\delta_{rl}+\arctan\left(\frac{v_{y}-\dot{\psi }a}{v_{x}-\dot{\psi }d}\right)\approx -\delta_{fr}+\frac{v_{y}-\dot{\psi }a}{v_{x}-\dot{\psi }d}\\ \alpha_{rr}=-\delta_{rr}+\arctan\left(\frac{v_{y}-\dot{\psi }a}{v_{x}+\dot{\psi }d}\right)\approx -\delta_{rr}+\frac{v_{y}-\dot{\psi }a}{v_{x}+\dot{\psi }d} \end{array}\right.$$

Then, the lateral wheel slip is expressed as

(12) $$\sigma_{yi}=\frac{v_{wxi}}{R_{i}\omega_{i}}\tan\alpha_{i},$$

and we have the resultant slip

(13) $$\sigma_{i}=\sqrt{\sigma_{xi}^{2}+\sigma_{yi}^{2}}$$

### 2.3. Driver Model

As to calculate the steering wheel input *δ_sw_*, a standard preview-based controller in CarSim is used as a driver model, which minimized the vehicle deviations from the desired path for a given preview time. Further, the desired yaw motion is generated through a reference model, which describes the ideal vehicle responses based on vehicle speed and *δ_sw_*, in the form of

(14) $$\frac{\omega_{z,des}\left(s\right)}{\delta_{sw}\left(s\right)}=\frac{k_{r}{\bar{\omega }}_{z,des}}{\left(T_{1}s+1\right)\left(T_{2}s+1\right)},$$

(15) $${\bar{\omega }}_{z,des}=\frac{v_{x}}{\left(a+b\right)+\frac{\left(bC_{r}-aC_{f}\right)mv_{x}^{2}}{2C_{r}C_{f}\left(a+b\right)}}$$

where *k_r_* denotes the gain of the reference model. In this work, we set *k_r_* = 0.05. *T*_1_ and *T*_2_ are tunning parameters. *C_f_* and *C_r_* represent the lateral stiffness of a single front/rear tire. Desired lateral velocity *v_y,des_* is set to zero to avoid unnecessary tire lateral slip. Since the vehicle rollover can easily lead to fatal traffic accidents, it is necessary to prevent it through braking under rollover propensity quantitatively described by a Rollover Index (RI) that depends on the vehicle lateral acceleration and body roll angle [10]. When the index reaches the target warning value *RI_th_*, brake control is adopted to prevent the vehicle from rolling over dangers. The RI is in the form of

(16) $$\left\{\begin{array}{l} RI=C_{1}\left(\frac{\left|\varphi_{t}\right|{\dot{\varphi }}_{th}+\varphi_{th}\left|{\dot{\varphi }}_{t}\right|}{\varphi_{th}{\dot{\varphi }}_{th}}\right)+C_{2}\left(\frac{\left|a_{y}\right|}{a_{y,c}}\right)+\left(1-C_{1}-C_{2}\right)\frac{\left|\varphi_{t}\right|}{\sqrt{\varphi_{t}^{2}+{\dot{\varphi }}_{t}^{2}}},\;\mathrm{if}\;\varphi \left(\dot{\varphi }-k\varphi \right)\geq 0\\ RI=0,\;\mathrm{if}\;\varphi \left(\dot{\varphi }-k\varphi \right)<0 \end{array}\right.$$

When the RI reaches the defined target value *RI_th_*, we control the lateral acceleration by braking to reduce the RI down to *RI_th_*, and the desired lateral acceleration is described as

(17) $$\left|a_{y,des}\right|=\frac{a_{y,c}}{C_{2}}[RI_{des}-C_{1}\left(\frac{\left|\varphi_{t}\right|{\dot{\varphi }}_{th}+\varphi_{th}\left|{\dot{\varphi }}_{t}\right|}{\varphi_{th}{\dot{\varphi }}_{th}}\right)-\left(1-C_{1}-C_{2}\right)\frac{\left|\varphi_{t}\right|}{\sqrt{\varphi_{t}^{2}+{\dot{\varphi }}_{t}^{2}}}]$$

Considering the relationship between vehicle lateral acceleration and driving speed

(18) $$a_{y,des}={\dot{v}}_{y}+v_{x,des}\omega_{z},$$

(19) $$a_{y}={\dot{v}}_{y}+v_{x}\omega_{z}$$

It follows that

(20) $$v_{x,des}=v_{x}+\frac{1}{\omega_{z}}\left(a_{y,des}-a_{y}\right)$$

Otherwise, we consider the desired longitudinal speed as a constant cruising value when the RI was controlled within a reasonable range. Then the desired vehicle speed becomes

(21) $$v_{x,des}=v_{x,cons}$$

## 3. Design of the Control System

**Figure 3:**
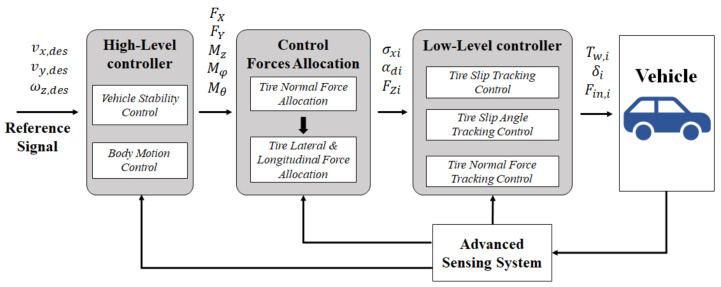
The architecture of unified vehicle dynamics control.

For a UCC design with all four wheels having independent torque, suspension, and steering-by-wire functions, the vehicle works as a redundantly actuated system. A hierarchical control structure shown in Figure 3 is presented to coordinate different control subsystems by the allocation of tire forces.

In this scheme, the high-level controller determines the resultant force/moment affected on the vehicle according to the control targets, including both the vehicle lateral stability and the spatial body motion (roll and pitch). Regarding the desired body motion dynamics, the target values of lateral and longitudinal transfer among the normal forces of every tire can be obtained. With the allocated normal forces through active suspension control, the CA algorithm distributes the resultant lateral and longitudinal forces to every wheel, which are finally realized through the closed-loop tracking control methods by considering the nonlinear tire model. In this work, we ignore the tire transient dynamics and assume that the desired tire longitudinal/lateral forces are well tracked through the variation of wheel steering angle *δ_i_* and the driving/braking torque *T_w_*_,*i*_. The steering angle of each wheel can be obtained through (11) with the desired value of wheel slip angle. According to the desired wheel longitudinal forces, *T_w_*_,*i*_ are calculated as,

(22) $$T_{w,i}=J_{w,i}{\dot{\omega }}_{w,i}+F_{x,i}r_{w,i},$$

where *J_w_*_,*i*_ include the wheel-side inertia. *r_w_*_,*i*_ and *ω_w_*_,*i*_ are the radius and the angular speed of the wheels, respectively. The values of *δ_i_* and *T_w_*_,*i*_ are treated as the inputs of the vehicle model in simulations. We also assume that all necessary quantitires in the control design can be practically measured or estimated.

### 3.1. High-Level Robust Controller Design

Parameter uncertainty is a common issue that requires robustness in control design. Compared with the actual vehicle model, common parameters with uncertainty include vehicle mass *m*, the inertia of moment *I_xx_*, *I_yy_*, *I_zz_*, rolling center position *e_r_*, pitching center position *e_p_*, unmodeled dynamics such as suspension dynamics, and disturbance like wind gust and road roughness. Addressing the vehicle non-linearity and mentioned system uncertainties, a sliding mode controller is proposed for system robustness based on the simplified dynamics. If we define the system states as

$$x_{i}=[v_{x},v_{y},\dot{\psi },\dot{\varphi },\dot{\theta }]$$

The vehicle system can be given in state-space form as

(23) $$\left\{\begin{array}{l} {\dot{x}}_{1}=x_{2}x_{3}-\frac{1}{m}F_{r}-\frac{m_{s}}{m}e_{r}{\dot{x}}_{3}x_{4}+\frac{1}{m}u_{1}+\Delta_{1}\\ {\dot{x}}_{2}=-x_{1}x_{3}+\frac{m_{s}}{m}e_{r}{\dot{x}}_{4}+\frac{1}{m}u_{2}+\Delta_{2}\\ {\dot{x}}_{3}=\frac{1}{I_{zz}}u_{3}+\Delta_{3}\\ {\dot{x}}_{4}=\frac{a_{y}\left[m_{s}\left(h_{r}+e_{r}\right)+m_{u}h_{a}\right]}{I_{xx}}+\frac{m_{s}ge_{r}\varphi }{I_{xx}}+\frac{1}{I_{xx}}u_{4}+\Delta_{4}\\ {\dot{x}}_{5}=-\frac{a_{x}\left[m_{s}\left(h_{p}+e_{p}\right)+m_{u}h_{a}\right]}{I_{yy}}+\frac{m_{s}ge_{p}\theta }{I_{yy}}+\frac{1}{I_{yy}}u_{5}+\Delta_{5} \end{array}\right.$$

where ∆*_i_* stand for unmodeled dynamics. The control inputs are defined as

$$\left(u_{1},u_{2},u_{3},u_{4},u_{5}\right)=\left(F_{X},F_{Y},M_{z},M_{\varphi },M_{\theta }\right)$$

These equations can be treated as five single-input-single-output systems, then the control inputs *u*_1_,…,*u*_5_ become decoupled. Next, we select the velocity error as the sliding surface for state *x*_1_, *x*_2_, and *x*_3_ for the path following purpose. Considering that the body roll and pitch motions should not be avoided completely for tire contact stability, we design dynamics sliding surfaces for the other two states.

(24) $$\left\{\begin{array}{l} S_{n}=e_{n}=x_{n}-x_{n,des},\;n=1,2,3\\ S_{n}=x_{4}+C_{\varphi }\varphi ,\;n=4\\ S_{n}=x_{5}+C_{\theta }\theta ,\;n=5 \end{array}\right.$$

In this paper, we turn the coefficients as *C_φ_* = *C_θ_* = 1. For each channel, the Lyapunov function candidate is in the form of

(25) $$V_{n}=\frac{1}{2}S_{n}^{2}$$

To achieve the attractive behavior of the sliding surface in a finite time period, it follows that

(26) $${\dot{V}}_{n}=S_{n}{\dot{S}}_{n}\leq -\eta_{n}\left|S_{n}\right|$$

where the value of *η_n_*(>0) decides the speed of sliding surface convergence. The attractive equations can be given as

(27) $${\dot{S}}_{n}=-K_{n}Sign\left(S_{n}\right)$$

Taking channel one as example, we have

(28) $$x_{2}x_{3}-\frac{F_{r}}{m}-\frac{m_{s}}{m}e_{r}{\dot{x}}_{3}x_{4}+\frac{1}{m}u_{1}+\Delta_{1}-{\dot{x}}_{1,des}=-K_{1}Sign\left(S_{1}\right)$$

In practical use, some system parameters remain unknown to control design. Thus, the desired control efforts can only be expressed based on the nominal values, such as

(29) $$u_{1}=\bar{m}[-x_{2}x_{3}+\frac{F_{r}}{\bar{m}}+\frac{{\bar{m}}_{s}}{\bar{m}}{\bar{e}}_{r}{\dot{x}}_{3}x_{4}+{\dot{x}}_{1,des}-{\bar{\Delta }}_{1}-K_{1}Sign\left(S_{1}\right)]$$

Substituting this value into (26), it follows that

(30) $${\dot{V}}_{1}=S_{1}\left\{\left(1-\frac{\bar{m}}{m}\right)x_{2}x_{3}+\left(\frac{{\bar{m}}_{s}{\bar{e}}_{r}}{m}-\frac{m_{s}e_{r}}{m}\right){\dot{x}}_{3}x_{4}+\left(\frac{\bar{m}}{m}-1\right){\dot{x}}_{1,des}+\left(\Delta_{1}-\frac{\bar{m}}{m}{\bar{\Delta }}_{1}\right)-\frac{\bar{m}}{m}K_{1}Sign\left(S_{1}\right)\right\}$$

Here, we select the nominal values as

(31) $$\left\{\begin{array}{l} \bar{m}=\sqrt{m_{\min}m_{\max}},\;\beta_{m}^{-1}\leq \frac{\bar{m}}{m}\leq \beta_{m}\\ {\bar{m}}_{s}=\sqrt{m_{s,\min}m_{s,\max}},\;\beta_{ms}^{-1}\leq \frac{{\bar{m}}_{s}}{m_{s}}\leq \beta_{ms}\\ {\bar{e}}_{r}=\sqrt{e_{r,\min}e_{r,\max}},\;\beta_{er}^{-1}\leq \frac{{\bar{e}}_{r}}{e_{r}}\leq \beta_{er} \end{array}\right.$$

where,

$$\beta_{m}=\sqrt{\frac{m_{\max}}{m_{\min}}},\beta_{ms}=\sqrt{\frac{m_{s,\max}}{m_{s,\min}}},\beta_{er}=\sqrt{\frac{e_{r,\max}}{e_{r,\min}}}$$

Then all factors in (30) have corresponding upper bounds

(32) $$\left\{\begin{array}{l} \left|1-\frac{\bar{m}}{m}\right|\leq \max\left\{\left|1-\beta_{m}^{-1}\right|,\left|1-\beta_{m}\right|\right\}={\bar{\beta }}_{11}\\ \left|\frac{{\bar{m}}_{s}{\bar{e}}_{r}}{m}-\frac{m_{s}e_{r}}{m}\right|\leq \max\left\{\left|1-\beta_{ms}^{-1}\beta_{er}^{-1}\right|,\left|1-\beta_{ms}\beta_{er}\right|\right\}e_{r,\max}={\bar{\beta }}_{12}\\ \left|\Delta_{1}-\frac{\bar{m}}{m}{\bar{\Delta }}_{1}\right|\leq \max\left\{\left|\Delta_{1,\max}\right|,\left|\Delta_{1,\min}\right|\right\}+\beta_{m}\left|{\bar{\Delta }}_{1}\right|={\bar{\beta }}_{13} \end{array}\right.$$

Due to the assumption that all the vehicle states and their derivates are physically upper bounded, Equation (30) becomes

(33) $${\dot{V}}_{1}\leq \left[{\bar{\beta }}_{11}{\left|x_{2}x_{3}\right|}_{m}+{\bar{\beta }}_{12}{\left|{\dot{x}}_{3}x_{4}\right|}_{m}+{\bar{\beta }}_{11}{\left|{\dot{x}}_{1,des}\right|}_{m}+{\bar{\beta }}_{13}-\beta_{m}^{-1}K_{1}\right]\left|S_{1}\right|\leq -\eta_{1}\left|S_{1}\right|$$

In order to achieve the convergence inequality, it is then sufficient to have

(34) $$K_{1}\geq \beta_{m}\left({\bar{\beta }}_{11}{\left|x_{2}x_{3}\right|}_{m}+{\bar{\beta }}_{12}{\left|{\dot{x}}_{3}x_{4}\right|}_{m}+{\bar{\beta }}_{11}{\left|{\dot{x}}_{1,des}\right|}_{m}+{\bar{\beta }}_{13}+\eta_{1}\right)$$

To avoid the chattering effects caused by the switching function, it is replaced by a saturation function, which is a continuous approximation with the thickness of *ϕ*_1_, and then the control law of channel 1 becomes

(35) $$u_{1}=\bar{m}[-x_{2}x_{3}+\frac{1}{2\bar{m}}CAx_{1}^{2}+\frac{{\bar{m}}_{s}}{\bar{m}}{\bar{e}}_{r}{\dot{x}}_{3}x_{4}+{\dot{x}}_{1,des}-{\bar{\Delta }}_{1}-K_{1}Sat\left(\frac{S_{1}}{\phi_{1}}\right)]$$

Through the same process, the control laws for other sliding surfaces are

(36) $$u_{2}=\bar{m}\left[x_{1}x_{3}+\frac{{\bar{m}}_{s}}{\bar{m}}{\bar{e}}_{r}{\dot{x}}_{4}+{\dot{x}}_{2,des}-{\bar{\Delta }}_{2}-K_{2}Sat\left(\frac{S_{2}}{\phi_{2}}\right)\right]$$

(37) $$u_{3}={\bar{I}}_{zz}\left[{\dot{\omega }}_{z,des}-{\bar{\Delta }}_{3}-K_{3}Sat\left(\frac{S_{3}}{\phi_{3}}\right)\right]$$

(38) $$u_{4}=-a_{y}\left[{\bar{m}}_{s}\left({\bar{h}}_{r}+{\bar{e}}_{r}\right)+{\bar{m}}_{u}h_{a}\right]-{\bar{m}}_{s}{\bar{e}}_{s}g\varphi +{\bar{I}}_{xx}\left[-C_{\varphi }x_{4}-{\bar{\Delta }}_{4}-K_{4}Sat\left(\frac{S_{4}}{\phi_{4}}\right)\right]$$

(39) $$u_{5}=a_{x}\left[{\bar{m}}_{s}\left({\bar{h}}_{p}+{\bar{e}}_{p}\right)+{\bar{m}}_{u}h_{a}\right]-{\bar{m}}_{s}{\bar{e}}_{p}g\theta +{\bar{I}}_{yy}\left[-C_{\theta }x_{5}-{\bar{\Delta }}_{5}-K_{5}Sat\left(\frac{S_{5}}{\phi_{5}}\right)\right]$$

For every channel, by choosing the *K*_n_ to be sufficiently large, the convergence inequalities can be guaranteed, and the sliding surfaces are designed to be attractive. In this paper, we choose *K*_1_ = 0.02, *K*_2_ = 0.2, *K*_3_ = 0.01, *K*_4_ = 0.1, and *K*_5_ = 0.1. Through the appropriate CA method, the high-level control efforts *u*_1_-*u*_5_ are distributed into four wheels. However, we cannot always achieve these control efforts considering that the tire forces are generated through the contact behavior between tires and road and are physically bounded.

### 3.2. Control Allocation Algorithm

The hierarchical control algorithm with a redundant set of actuators frequently contains a high-level controller to generate virtual control efforts and a control allocation algorithm to coordinate the actuators to produce the desired control values together. If the control efforts require forces beyond the capabilities of the actuators due to saturation or physical limitations, the CA algorithm should be able to lower its performance and search for a control input vector that minimized the error. Additionally, redundant actuators can be utilized to provide fault tolerance for safety-critical conditions such as normal force losing. For real-time applications, the computational effort is also an essential property for choosing the CA algorithm. In this viewpoint, we applied a fixed-point CA method to solve the CA problem in constraint system control. Based on the nonlinear vehicle and tire models, the generalized force/moment can be expressed using a set of nonlinear functions of control variables

(40) $$u_{d}=F\left(\lambda ,U\right),$$

where, $\lambda ={\left[F_{zi},\mu_{i},\delta_{i}\right]}^{T}$ is a 12 × 1 vector configurating the CA problem and remains invariable during a CA computation step. *¦Ì_i_* is the friction coefficient between road and tires. $U={\left[\alpha_{i},\kappa_{i}\right]}^{T}$ is a 8 × 1 control vector of slip angle and slip ratio of each tire. **u**_d_ denotes the desired generalized force, which can be rewritten using a first-order linearizing expression about an operating point,

(41) $$\begin{array}{l} F\left(\lambda ,U\right)\approx F\left(\lambda ,U_{k-1}\right)+\frac{\partial F}{\partial U}\left(\lambda ,U_{k-1}\right)\cdot \left(U_{k}-U_{k-1}\right)\Rightarrow \\ F\left(\lambda ,U\right)-F\left(\lambda ,U_{k-1}\right)+\frac{\partial F}{\partial U}\left(\lambda ,U_{k-1}\right)\cdot U_{k-1}=\frac{\partial F}{\partial U}\left(\lambda ,U_{k-1}\right)\cdot U_{k}=B_{F}\cdot U_{k} \end{array}$$

where, **B***_F_* is a Jacobian matrix defined by the configurating vector and the control vector of the last step, which describes the sensitivity of the control vector to desired value vector **u′***_d_*, which is in the form of

(42) $${u^{\prime }}_{d}=u_{d}-F\left(\lambda ,U_{k-1}\right)+\frac{\partial F}{\partial U}\left(\lambda ,U_{k-1}\right)\cdot U_{k-1}$$

Then, Equation (41) becomes

(43) $${u^{\prime }}_{d}\approx B_{F}\cdot U_{k}$$

The CA algorithm minimized the following objective function, including the CA errors and control effort. The optimization criteria can be given as

(44) $$\min J=\frac{1}{2}\left(1-\varepsilon \right)\Gamma^{T}W_{e}\Gamma +\frac{1}{2}\varepsilon U^{T}W_{U}U$$

where Γ = (**B***_F_***U** − **u′***_d_*) is the control error vector, and the variation of control value **U**∈[**U***_L_*, **U***_U_*] is restrained by actuator rate limits and achievable value ranges. The two bounds are decided by

(45) $$\left\{\begin{array}{l} U_{L}=\max[U_{\min},U_{k-1}-\tau \cdot r_{\max}]\\ U_{U}=\min[U_{\max},U_{k-1}+\tau \cdot r_{\max}] \end{array}\right.$$

where, *τ* represents the sampling time and **r**_max_ is the maximum rates of variable values. This constraint helps to ensure the process stability. *ε* is a number to balance the weight between control efforts error and actuation cost, which is chosen as 0.5. Suppose that we want to minimize *J*:R^8^→*R*, a gradient descent algorithm has the iterative step

(46) $$U_{i+1}=U_{i}-\eta \nabla J\left(U_{i}\right)$$

∇*J*(**U**) is the derivative of *J* at **U**. Substituting the derivative of *J* into this equation. It follows that

(47) $$\left\{\begin{array}{l} U_{i+1}=sat\left[\left(1-\varepsilon \right)\eta B_{F}^{T}W_{e}{u^{\prime }}_{d}-\left(\eta T-I\right)U_{i}\right]:=I\left(U_{i}\right)\\ T=\left(1-\varepsilon \right)B_{F}^{T}W_{e}B_{F}+\varepsilon W_{U} \end{array}\right.$$

where the step length parameter *η* is set to *η* = 1/‖**T**‖*_F_*, with ‖*·*‖*_T_* being the Frobenius norm of a matrix, which is more computationally efficient than induced norms. The saturation function sat can cut the elements of the control vector **U***_(i+_*_1*)*_ at their limits. If *J* is convex and the operator *I* is convergent, then a point **U*_k_*** is a fixed point of *I* if and only if ∇*J*(**U*_k_***) = 0, so if and only if **U*_k_*** minimizes *J*, with the expression

(48) $$U_{k}=I\left(U_{k}\right)$$

### 3.3. Tire Normal Forces Robust Tracking Control

Based on the mentioned tire models, the boundaries of friction ellipses are directly affected by the normal tire forces, which are the control vectors of the high-level strategy for inhibiting undesired vehicle motion. The tracking control based on the active suspension system is a challenging task because of the following reasons.

(1) In practical use, the wheel motions are under the effect of road roughness, especially considering the high unsprung mass introduced by the electric propulsion system, such as the in-wheel motor. This kind of motion instability will cause the inaccuracy of tire load tracking.
(2) Quarter car model is the classic model which was widely utilized in suspension analysis and control synthesis. However, the spatial kinematics and dynamics considering the suspension geometry are relatively complicated, leading to the inaccuracy and uncertainty of model parameters. Though some analytic model of suspension geometry is presented and realized in simulation [33], the complex computation makes them less efficient in real-car implements.
(3) The active suspension control algorithm is always a trade-off between the vehicle ride comfort and tire-road adhesion stability, which is hard to be optimized simultaneously. Moreover, the tracking control requirement makes the problem more complex and increases the difficulty of controller design.

**Figure 4:**
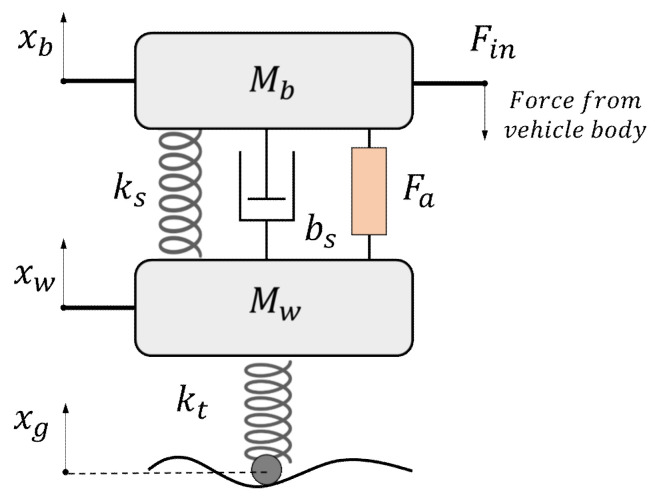
One-fourth active suspension model with body inertia force.

In this work, we used the traditional quarter car model and H-∞ robust control method to solve the modeling uncertainty and parameter variation problem. The model concept is illustrated through Figure 4, with *M_b_* as the one-fourth sprung mass, *M_w_* as the one-fourth unsprung mass, *k_s_* as the equivalent suspension stiffness, *b_s_* as the equivalent suspension damping, and *k_t_* as the tire stiffness. The force from the vehicle body *F_in_* is a known input. *F_a_* is the active force returned by the active suspension controller.

Based on the traditional quarter car model, an extra equivalent body inertia force *F_in_* is imposed upon the sprung mass to reflect the coupling effect between the quarter model and the full-vehicle model. The value of *F_in_* is supposed to be the desired normal tire force. Due to suspension geometry and ride comfort requirements, the equivalent suspension stiffness and damping are actually not constant values, which is treated as system uncertainties. The body inertia force and road profile are the outside disturbances to the system. However, we can still use their nominal values to establish the model. The states of the controlled system are defined as $\left[x_{1},x_{2},x_{3},x_{4}\right]=\left[x_{w},{\dot{x}}_{w},x_{b},{\dot{x}}_{b}\right]$, and generalized system inputs are $\left[u_{1},u_{2},u_{3}\right]=\left[F_{in},x_{g},F_{a}\right]$.

Then, the vertical dynamics of suspension can be given as

(49) $$\left\{\begin{array}{l} {\dot{x}}_{2}=-\left(\frac{k_{s}+k_{t}}{M_{w}}\right)x_{1}-\frac{b_{s}}{M_{w}}x_{2}+\frac{k_{s}}{M_{w}}x_{3}+\frac{b_{s}}{M_{w}}x_{4}+\frac{k_{t}}{M_{w}}u_{2}-\frac{1}{M_{w}}u_{3}\\ {\dot{x}}_{4}=\frac{k_{s}}{M_{b}}x_{1}+\frac{b_{s}}{M_{b}}x_{2}-\frac{k_{s}}{M_{b}}x_{3}-\frac{b_{s}}{M_{b}}x_{4}-\frac{1}{M_{b}}u_{1}+\frac{1}{M_{b}}u_{3} \end{array}\right.$$

**Figure 5:**
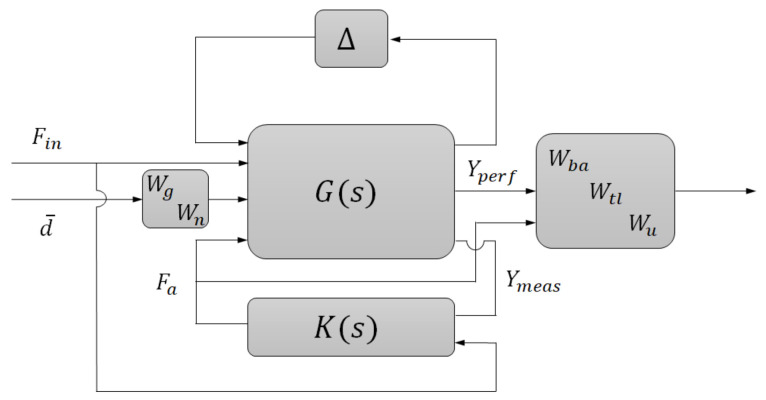
Robust tracking control of tire contact force.

The system performance index is selected as

(50) $$Y_{perf}=\left[{\ddot{x}}_{b},e_{n},F_{a}\right]$$

where the sprung mass acceleration is the index for ride comfort, and *e_n_* = *F_in_* − *k_t_*(*x_g_* − *x_w_*) expresses the normal force control error, defined as the tire contact stability index. *F_a_* stands for the active suspension actuator force. The system output includes the performance index as well as the signals to be measured for feedback control, which may contain the body (unsprung mass) acceleration (BA) and tire dynamic load (DL). We use the linear fractional transformation method to define an extended state-space model to deal with the parameter uncertainties. The closed-loop system diagram is described in Figure 5 [34].

**Figure 6:**
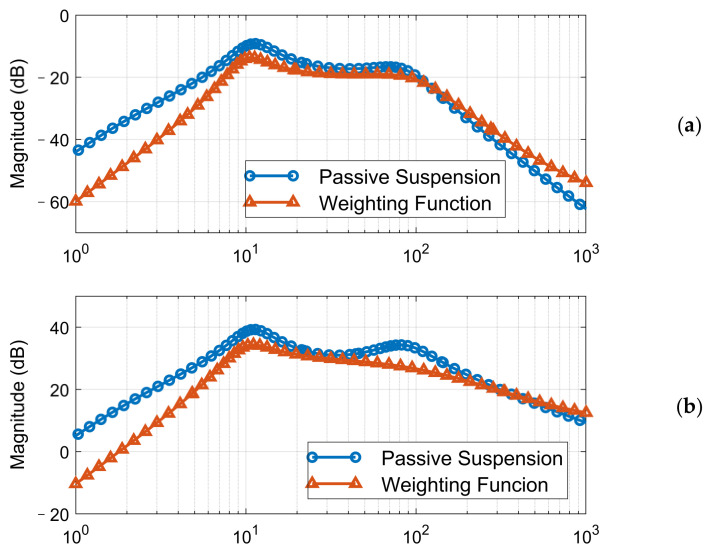
The inverse of system performance index weighting functions: (**a**) vertical acceleration; (**b**) tire dynamic load.

The weighting functions of system performance index *W_ba_*, *W_tl_*, and control output and *W_u_*, which define the target performance in the frequency domain, should be well tuned to acquire a stable and satisfactory controller. Here, we design and tune the weighting function according to the open-loop suspension performance, and the amplitude-frequency characteristics of BA and DL are compared with the inverse of the weighting function *W_ba_* and *W_tl_* in Figure 6. Furthermore, since the desired characteristic of BA and DL is hard to achieve simultaneously, we use different control structures to achieve the vehicle comfort target and tire stability target:

(1) Controller 1: For the state of tire contact force cannot reflect the dynamic behavior of sprung mass, only the sprung mass acceleration was selected as the feedback signal of the comfort-orientated control scheme.
(2) Controller 2: In the tire-stability-orientated control scheme, both the unsprung mass acceleration and tire contact force work as the feedback signals, with the former signal reflecting the desired tire normal force and the latter one providing the real normal force. The sensor reflects the DL could be tire-pressure based, for example.

**Figure 7:**
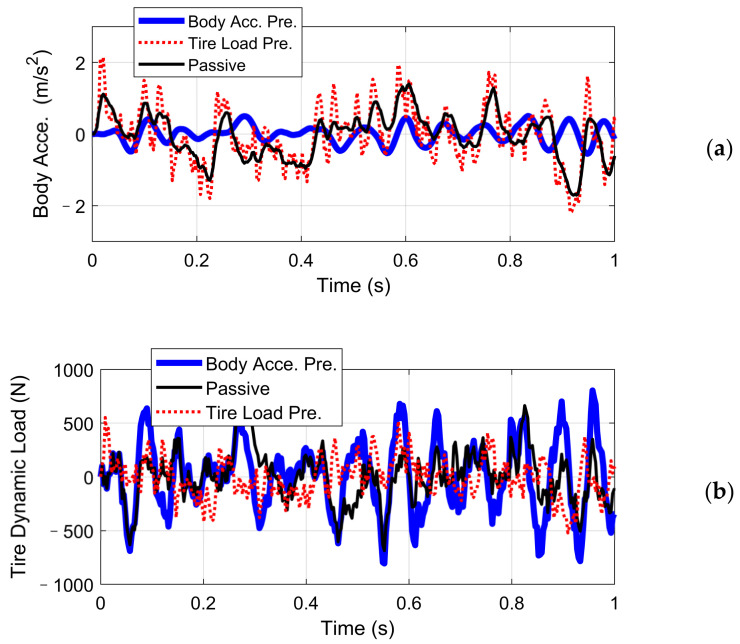
Performance of Controllers 1 and 2: (**a**) body acceleration; (**b**) tire dynamic load.

In order to compare the performance of the closed-loop system of controller 1 and controller 2, the state-space system models are established in the MATLAB environment, where the corresponding H-∞ controllers are synthesized through coupled Riccati equations [35]. Further, a sinusoidal input was built to test the tracking performance of controller 2. Simulation results concerning body acceleration and tire dynamic load in the time domain are shown in Figure 7. Their root mean square values are listed in Table 1 for comparison. An A-class uneven road is simulated by filtered white noise according to ISO8608, with the vehicle speed chosen as 120 km/h.

**Figure 8:**
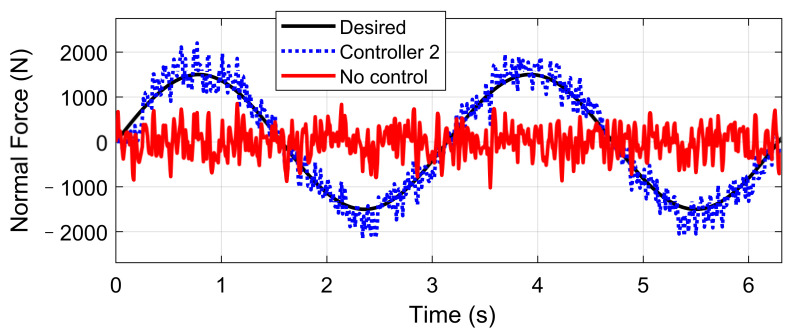
Normal force tracking of controller 2.

The simulation results illustrate that, under the comfort-oriented control, the body acceleration magnitude illustrated by the blue line was decreased, compared with the value without active control expressed by the black line. On the contrary, the average tire DL was increased. Reversely, the stability-oriented control inhibited the normal tire force tracking error but enlarged the body acceleration magnitude unavoidably. Results in Figure 8 indicated that controller 2 achieved good tracking performance under sinusoidal input signal with the suppression of road roughness disturbance.

Although we only consider the tracking control of tire vertical forces, energy consumption of the active suspension system cannot be neglected. The tradeoff between vibration suppression and energy consumption should be considered in practical applications, which could be realized by adjusting the weighting funcion *W_u_*.

## 4. Simulation Studies

In order to evaluate its overall performance, the UCC system with the application of the stability-oriented controller (controller 2) is implemented in a CarSim–Simulink co-simulation platform. The target vehicle is a B-class sports car. Its parameters are listed in Table 2. Compared with the traditional ESC approach, the dynamic performance and advantages of the proposed UCC method are interpreted through the following driving conditions.

### 4.1. High-Speed Double Lane-Changing (DLC) on a Rough Road Surface

**Figure 9:**
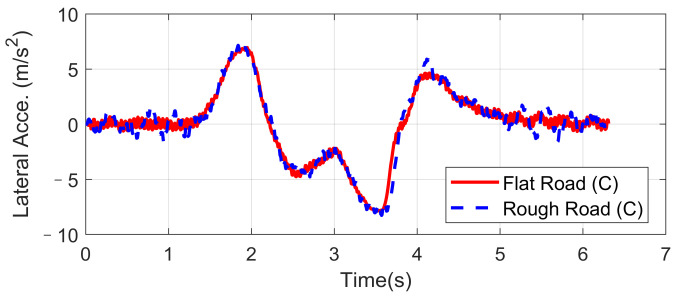
Lateral acceleration in high-speed DLC.

**Figure 10:**
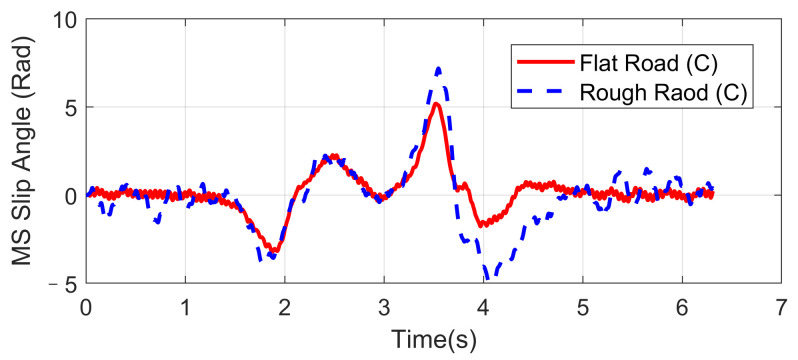
Vehicle slip angle in high-speed DLC.

**Figure 11:**
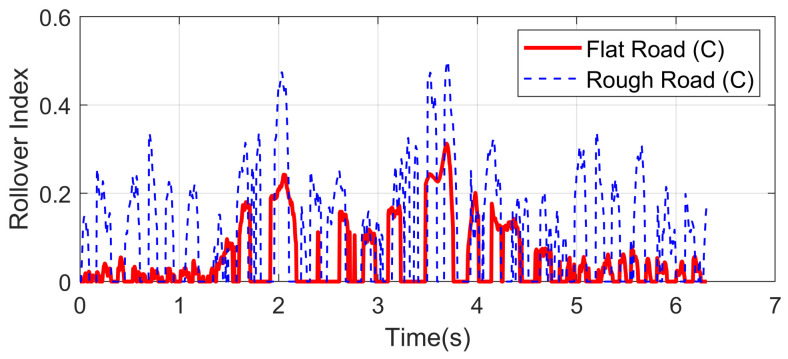
Rollover index in high-speed DLC.

**Figure 12:**
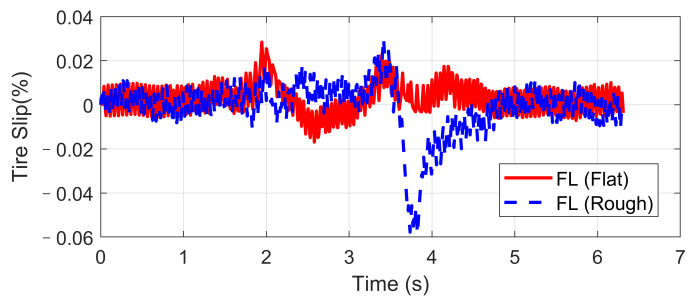
FL tire slip in high-speed DLC.

**Figure 13:**
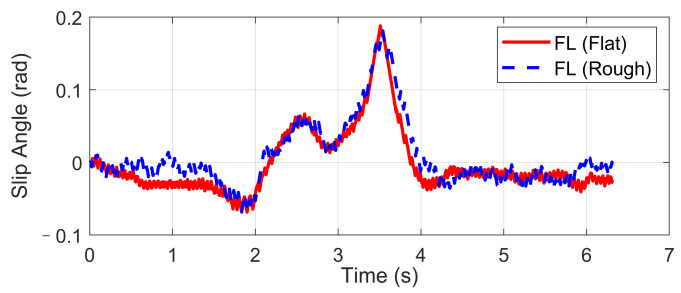
FL tire slip angle in high-speed DLC.

Double lane-changing is a standard test to evaluate the vehicle handling performance, which is usually done at a constant longitudinal speed. However, this maneuver becomes more challenging under a rough road surface at high speed because of the intensification of vertical vibration, and it is possible that the fluctuation of normal tire forces will influence the allocation accuracy of desired general longitudinal and lateral forces. In order to evaluate the dynamic performance of the proposed UCC system, a comparison simulation with the same controller was carried out based on the DLC test at 120 km/h speed under flat and rough road surfaces. The A-class uneven road profile is generated randomly. Since the effectiveness of active suspension control has been verified before, here we mainly focus on the lateral vehicle performances compared with the flat road situation. Simulations results are compared in Figure 9, Figure 10, Figure 11, Figure 12 and Figure 13.

The simulation verified that the CA method could generate desired resultant forces for the vehicle to complete the DLC maneuver under road roughness. Besides, the results in Figure 9, Figure 10 and Figure 11 demonstrate that the road disturbance generated slight undesired vehicle dynamics, including lateral and rolling motions. To investigate the effect of road roughness on the CA algorithm, the control values of the front left tire are compared in Figure 12 and Figure 13.

We can see from the results that the UCC method has a certain degree of robustness due to the sliding mode controller. However, in the critical points of DLC process (around 2 s and 3.5 s), the vehicle lateral acceleration and yaw rate approached their maximum values, and the tire forces were close to the boundary. In order to achieve the required lateral force, slip angles of four tires should not have a significant variation, as shown in Figure 12. In these critical time points, the normal force fluctuation led to an extra yaw moment, which is supposed to be compensated by the longitudinal vehicle forces. As can be seen in Figure 13, the tire slip variation in such time points was more significant than at other times, and this effect was even more severe in rough road conditions. Since in the proposed UCC strategy, the desired forces are evenly allocated into four tires, the situations of the other three tires were similar and will not be discussed to avoid redundant expression.

### 4.2. High-Speed Double Lane-Changing (DLC) on a Flat Road Surface (Compared with ESC)

**Figure 14:**
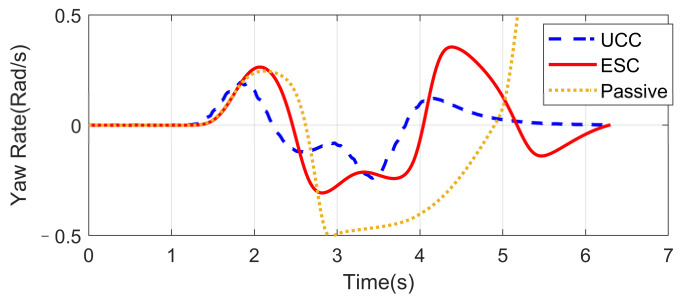
Yaw rate on flat road during high-speed DLC.

**Figure 15:**
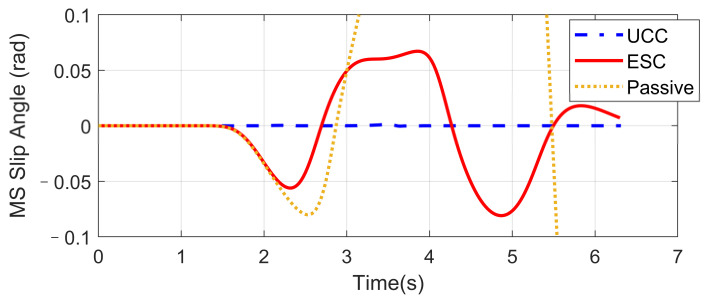
Vehicle slip angle on flat road during high-speed DLC.

**Figure 16:**
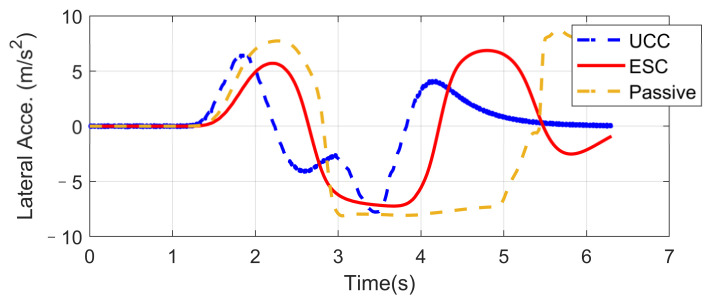
Lateral acceleration on flat road during high-speed DLC.

**Figure 17:**
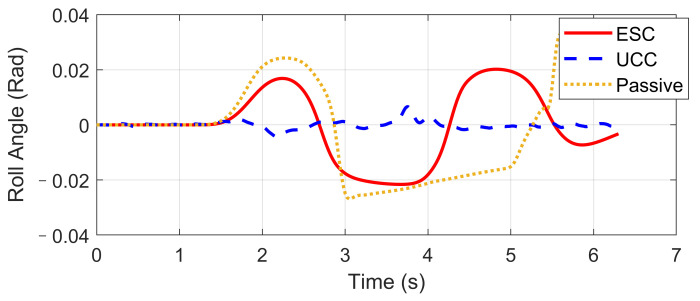
Body roll angle on flat road during high-speed DLC.

**Figure 18:**
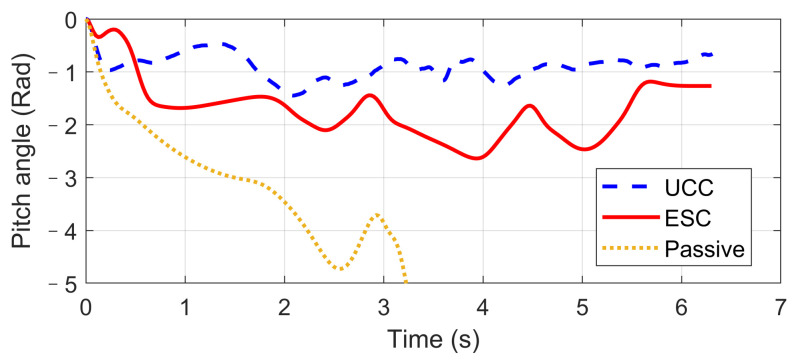
Body pitch angle on flat road during high-speed DLC.

**Figure 19:**
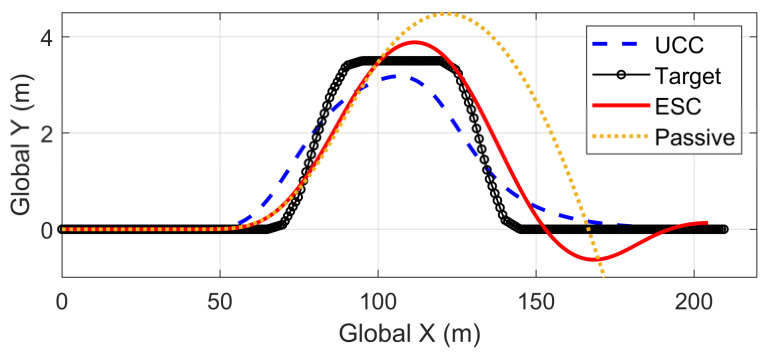
Vehicle path on flat road during high-speed DLC.

Based on the same DLC test, while the first scenario examined the UCC performance on the uneven road, this second one focused on the comparison with the traditional ESC method. The ESC system is an envelope stability controller based on direct yaw control, which can only control the longitudinal force distribution among four tires. The results in Figure 14, Figure 15, Figure 16, Figure 17, Figure 18 and Figure 19 explain the superiority of the proposed UCC system on lateral vehicle dynamics compared with the ESC control strategy. The vehicle failed to pass this scenario without active control.

Figure 14, Figure 15 and Figure 16 and Figure 19 show yaw rate, vehicle slip angle, lateral acceleration, and vehicle path, respectively, and illustrate that the UCC has better performance than the ESC with respect to the yaw stability. Therein the desired vehicle slip angle control was well done by the UCC control. This is due to the fact that the vehicle system with multiple actuators can make full use of the adhesion coefficient of each wheel to generate desired resultant force/moment. By comparing the vehicle rolling and pitch angles presented in Figure 17 and Figure 18, one can clearly see that the proposed UCC maintained the body altitude effectively, enhancing the vehicle ride comfort due to active suspension control.

### 4.3. High-Speed Fishhook Maneuver

**Figure 20:**
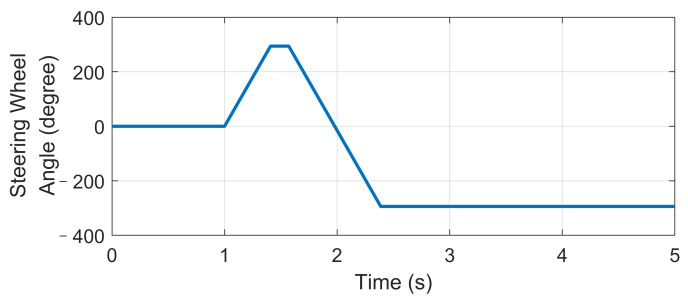
Steering angle in high-speed fishhook maneuver.

**Figure 21:**
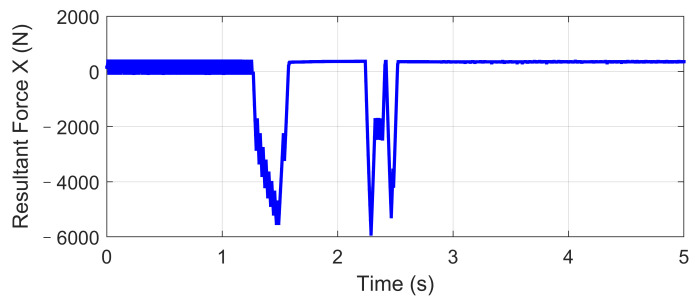
Longitudinal force in high-speed fishhook maneuver.

**Figure 22:**
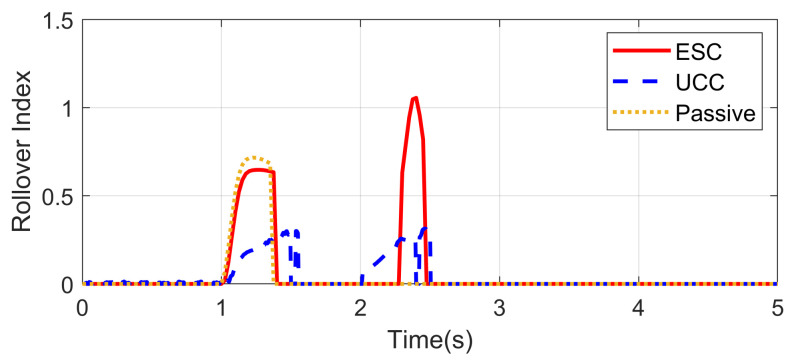
Rollover index in high-speed fishhook maneuver.

**Figure 23:**
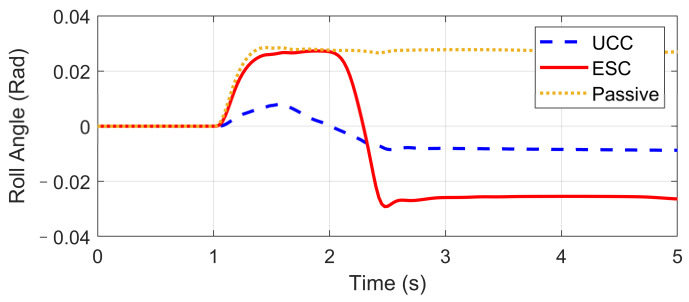
Body roll angle in high-speed fishhook maneuver.

**Figure 24:**
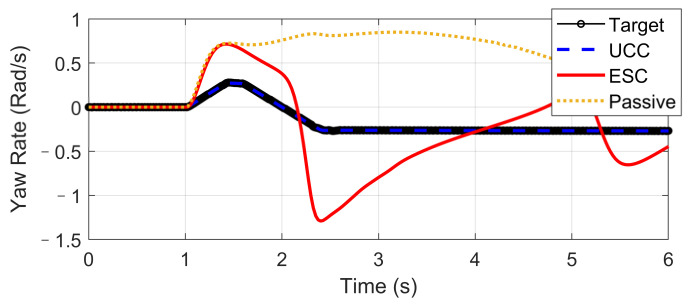
Yaw rate in high-speed fishhook maneuver.

**Figure 25:**
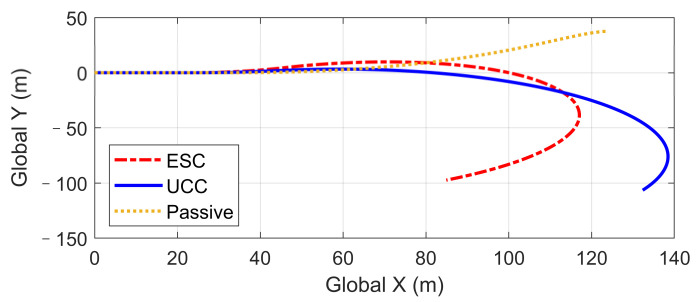
Vehicle global path in high-speed fishhook maneuver.

The Fishhook test is a dynamic test for predicting the dynamic rollover propensity. To begin the maneuver, the vehicle is driven in a straight line at the desired entrance speed, and then the driver releases the throttle and initiates the designed steering motion described in Figure 20. In this section, the steering maneuver of this test was adopted to verify the anti-rollover mechanism of the proposed UCC with the braking command triggered by the RI threshold set to 0.2. Figure 21, Figure 22, Figure 23, Figure 24 and Figure 25 shows the results at entrance speed 120 km/h concerning relative vehicle states. If no control strategy is applied, the vehicle will lose control early at around 1.5 s, as shown in the figures.

The result of vehicle RI clearly illustrates that, at time t = 1.5 and t = 2.3, when the steering angle reached its maximum value, the vehicle experienced the most apparent rollover propensity. Though the rollover did not happen actually due to the ESC control, the RI value still reached a dangerous level. The proposed UCC method can significantly reduce the RI value in the following two aspects.

(1) The body altitude control function of UCC reduced the vehicle roll angle, indicated in Figure 23.
(2) The braking force was triggered to inhibit the increase of lateral acceleration, which is shown in Figure 21.

We also observe from Figure 24 that the UCC method led to better dynamic performance and can achieve the desired yaw rate without losing stability. Additionally, the vehicle path curves shown in Figure 25 indicate that the undesired yaw moment generated by ESC control may promote the tendency of vehicle over-steering in high-speed curving situations, which can be avoided using the UCC with appropriate parameters tunning.

### 4.4. Tire Blow-Out in the Hard-Braking Process (Re-Configurable Control)

**Figure 26:**
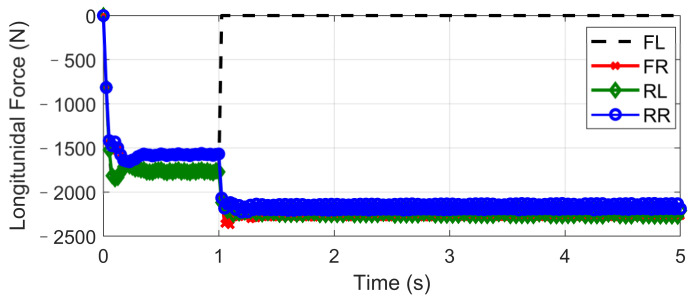
Longitudinal force in hard-braking.

**Figure 27:**
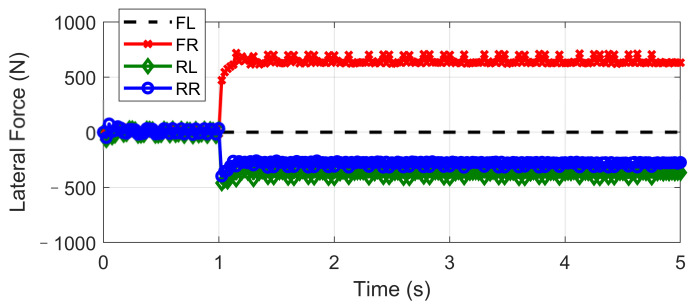
Lateral force in hard-braking.

**Figure 28:**
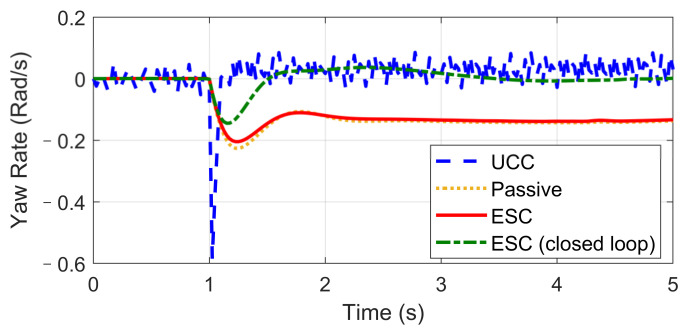
Yaw rate in hard-braking.

**Figure 29:**
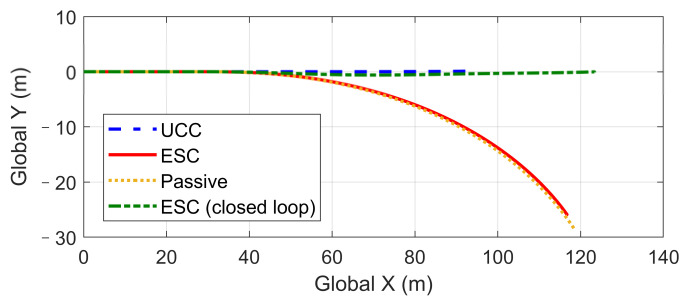
Vehicle path in hard-braking.

**Figure 30:**
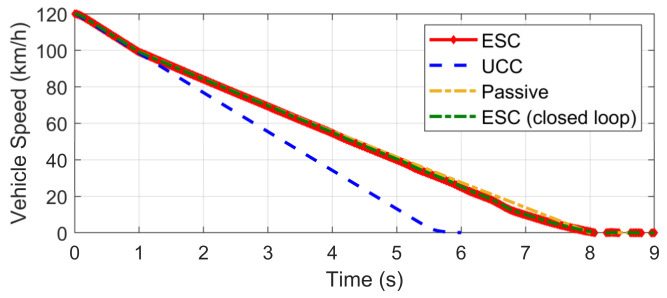
Vehicle speed in hard-braking.

This scenario is designed for the condition where one tire blew out during hard braking. The initial vehicle speed is 120 km/h and a hard braking command (about −0.6 g) is given at 0 s, and then at the end of the first second, the front-left tire blew out and lost its lateral and longitudinal forces. The steering input is kept as zero, which keeps in line with the actual reaction. Based on different control strategies, three different conditions are considered, including UCC, ESC control, and passive vehicle. Results concerning vehicle and tire states are provided in Figure 26, Figure 27, Figure 28, Figure 29 and Figure 30.

Curves in Figure 26 and Figure 27 described the re-allocation of tire longitudinal and lateral forces (in the body coordinate) after the front-left tire blew out through a reconfigurable UCC approach, keeping the desired resultant forces/moment unchanged. The vehicle yaw rate and path based on different control configurations are shown in Figure 28 and Figure 29. These results illustrate the extra yaw moment due to the tire blowing-out and the lane departure behavior it caused for both the ESC control and passive situations. However, the prompt intervention of driver steering input (closed-loop ESC in figures) can reduce the danger to some extent accompanied by ESC, but it is highly demanding for non-professional drivers to respond appropriately under such emergencies.

On the contrary, the UCC just required the driver to keep straight ahead. Additionally, it is worth noticing in Figure 30 that the traditional ESC control cannot compensate for the lost tire brake forces. Thus, the brake distance was increased. However, the UCC reconfigurable method was able to hold the initial braking deceleration and inhibit the interference of extra yaw moment due to the re-allocation of tire forces. The proposed UCC is the only strategy that is able to keep the driver’s intention for this emergency.

## 5. Conclusions

In this paper, a UCC strategy involving the vehicle yaw stability, body altitude, and tire contact stability was presented. A hierarchical control structure was adopted to realize the UCC, including high-level sliding mode control, fixed point CA, and a normal tire force robust tracking controller. Simulations of high-demanding driving situations concerning rough road surface, fast DLC, fishhook maneuver, and the tire blowing-out situation performed on a nonlinear full vehicle model have shown the effectiveness and advantages of the proposed control method. The following conclusions can be drawn:

(1) For a four-wheel independent drive–independent steering configuration, the proposed UCC method can effectively realize the tire planar force distribution and the desired vehicle motion, which proves to be a practical solution due to its simple feedback control rules and reconfigurable allocation.
(2) Considering the wheel vertical vibrations, we present a robust tire normal force tracking controller to address the tire contact stability issue. This work provides an innovative method for improving the vehicle driving stability and maintaining the desired body attitude simultaneously.

## Figures and Tables

**Table 1:** RMS values of body acceleration and tire dynamic load.

Suspension Performances	Passive Suspension (without Control)	Comfort-Orientated Control	Stability-Orientated Control
Body acceleration (m/s^2^)	0.6732	0.2795 (↓58%)	0.9067 (↑35%)
Tire dynamic load (N)	242.7421	377.5729 (↑56%)	204.7990 (↓16%)

**Table 2:** Vehicle parameters in the simulation.

Symbol	Description	Values and Units
*m*	Vehicle mass	1140 kg
*C_D_*	Aerodynamic drag coefficient	0.34
*a*	Distance of front wheel axle from C.G.	1.165 m
*b*	Distance of rear wheel axle from C.G.	1.165 m
*d*	Half of the wheel base	0.7405 m
*I_zz_*	Yaw inertia	996 kg m^2^
*m_s_*	Vehicle sprung mass	1020 kg
*k_s_*	Suspension stiffness	33,972 N/m
*b_s_*	Suspension damping	2000 N s/m
*k_t_*	Tire stiffness	200,000 N/m

## Abbreviations

UCC	Unified Chassis Control	ESC	Electric Stability Control
CG	Center of Gravity	RI	Rollover Index
BA	Body Acceleration	DL	Tire Dynamic Load
DLC	Doubel Lane Change	CA	Control Allocation
